# Clinching for sheet materials

**DOI:** 10.1080/14686996.2017.1320930

**Published:** 2017-05-31

**Authors:** Xiaocong He

**Affiliations:** ^a^ Innovative Manufacturing Research Centre, Faculty of Mechanical and Electrical Engineering, Kunming University of Science and Technology, Kunming, PR China

**Keywords:** Clinching, lightweight sheet material, clinching joinability, hybrid clinching, modified clinching, 10 Engineering and Structural materials, 303 Mechanical / Physical processing, 402 Multi-scale modeling

## Abstract

Latest developments in the clinching of sheet materials are reviewed in this article. Important issues are discussed, such as tool design, process parameters and joinability of some new lightweight sheet materials. Hybrid and modified clinching processes are introduced to a general reader. Several unaddressed issues in the clinching of sheet materials are identified.

## Graphical abstract

**Figure:**
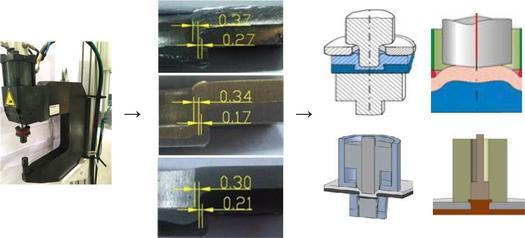

## 1. Introduction

Public demand for a sustainable use of resources has led to new design criteria for industrial products. There is an increasing need to design lightweight structures. Lightweight sheet materials and suitable joining methods are integral parts of lightweight structure manufacturing. A full evaluation of suitable joining techniques and their relative merits should take place prior to sheet materials selection [1–5].

**Figure 1.**
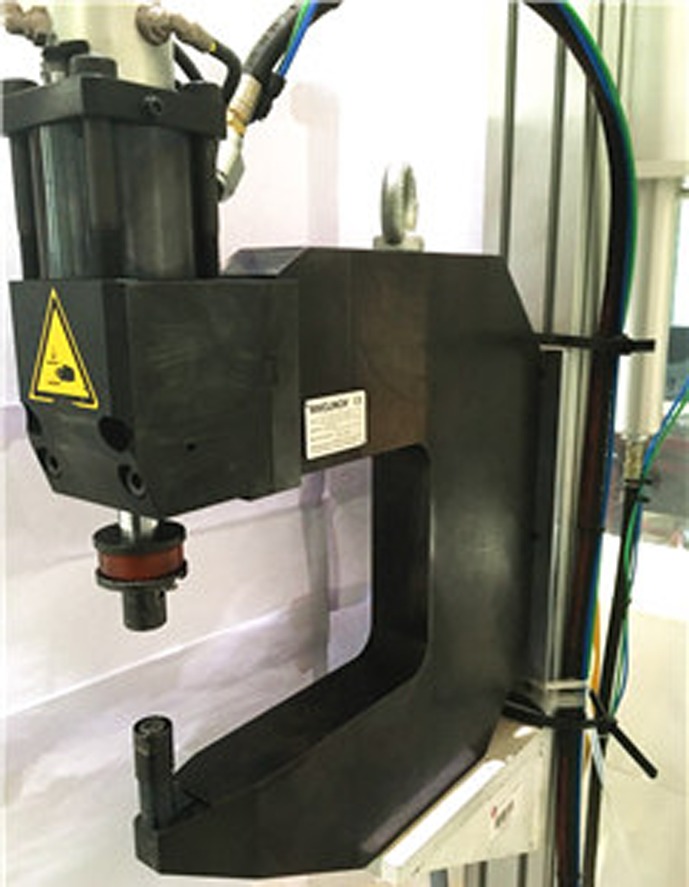
A typical clinching machine.

**Figure 2.**
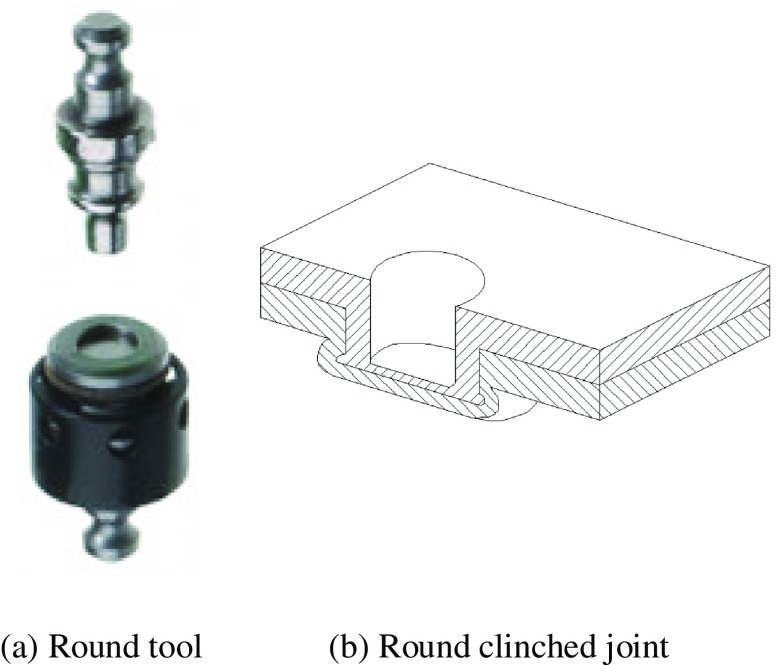
Round clinching.

**Figure 3.**
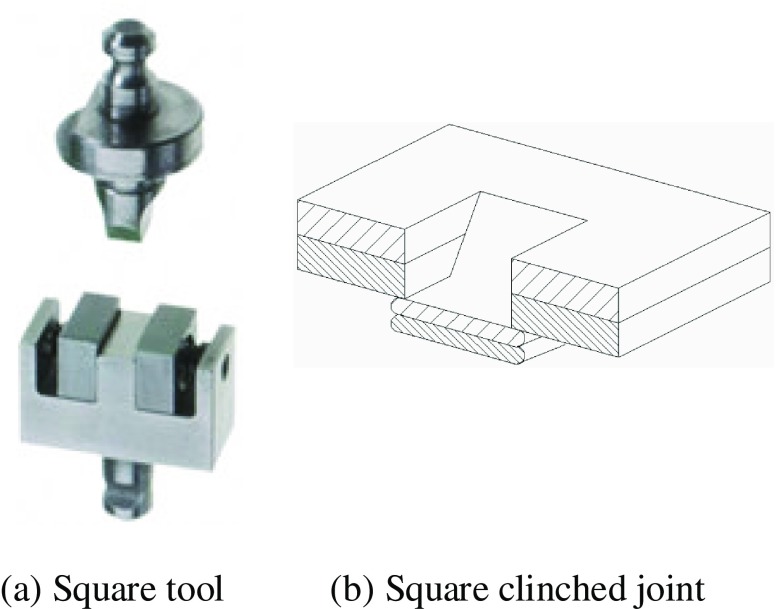
Square clinching.

**Figure 4.**
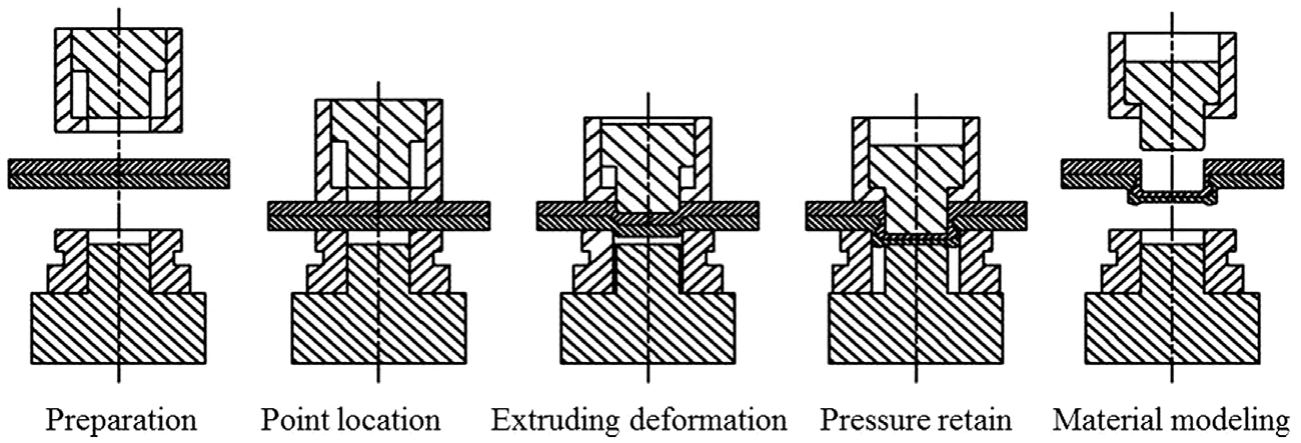
Principle of round clinching.

Clinching opens new possibilities of joining lightweight sheet materials in the assembly field of lightweight structure manufacturing [6–9]. The uninterrupted action of cold-forming produces the joint element at the clinched point directly out of the sheet material components. Lightweight sheet materials normally include low-density metals, such as magnesium and titanium, and nonmetals, such as plastics and various composites. Metal–metal combinations can be joined by conventional clinching, but some metal–nonmetal pairs can only be connected by hybrid clinching or modified clinching. Figure 1 shows a typical clinching machine. Depending on the tools used, clinching can be classified into round clinching and square clinching. Sheet materials are only deformed by round clinching, as shown in Figure 2. Both deformation and cutting of sheet will be required in square clinching, as shown in Figure 3. Thus the fatigue properties will be affected and the water tightness does not apply to square clinching. Both round clinching and square clinching are not recommended for brittle materials as mechanical clinching is generally a cold-forming process. The principle of round clinching is given in Figure 4 [10].

Compared to the more traditional sheet material joining methods, such as spot welding, spot friction stir welding, and self-piercing riveting, the main advantages of clinching include [6,7,11]:

(1) the ability to join multiple similar or dissimilar sheet materials;
(2) improved fatigue properties of the product;
(3) ease of process automation and monitoring; and
(4) environmental safety.

As with any sheet material joining technology, however, clinching has some disadvantages:

(1) relatively high force required for the forming process; and
(2) inapplicability to brittle sheet materials.

Though the first patent related to clinching was granted in 1897 [12], it is only in the last 30 years that clinching has significantly developed. Since presented at the 4th European Machine Tool Exhibition in Hannover in 1981, innovative clinching has constantly been under development. The first large scale use of clinching was at the Audi company in 1985 [13]. Some automobile manufacturers employ clinching in body assembly lines for its flexibility and reliability. Clinching has also been used in door manufacturing by Ford Motor Company since 1987 [14]. The original expanding clinching method was developed by Suzuki Motor Co. Ltd to join the aluminum outer tube of the bush for prevention action against galvanic corrosion [15].

The mechanical properties and corrosion resistance of clinched joints have been determined by testing, and compared with the corresponding values for welded joints since the 1980s [16–20]. The tests showed that good joints could be produced by the clinch method, and that they were in all respects comparable to spot welded joints. The quality of the joint depended above all on the type of clinch used, the adjustment of the equipment, and the orientation of the loading direction. If the possibilities for technical utilization and the economics of the two processes are compared, clinching is found to be more versatile than resistance spot welding, and is also suitable for use with combinations of different materials, while clinching also offers certain advantages with respect to both fixed and variable costs.

Several significant developments in clinching technology have been accompanied by theoretical and experimental investigations such as those at Technical University of Hamburg-Harburg (Germany) [21–25], the University of Paderborn (Germany) [26–30], the University of Edinburgh (UK) [31–35], Lappeenranta University of Technology (Finland) [36–40], KU Leuven, Technology Campus Ghent (Belgium) [41–45], Toyohashi University of Technology (Japan) [46–50], University of l’Aquila (Italy) [51–55], Xi’an Jiaotong University (China) [56–60], and Kunming University of Science and Technology (China) [61–65]. Significant progress has been made in the sheet materials clinching processes. However, no comprehensive review on sheet materials clinching processes has been reported so far.

Recent progress in clinching processes is reviewed in this paper. Important issues such as tool design and clinching processes are discussed. Clinching joinability of some new lightweight sheet material is introduced. Hybrid clinching processes and modified clinching processes are also introduced. Several unaddressed issues in the clinching of sheet materials are identified.

## 2. Clinching tool design

**Figure 5.**
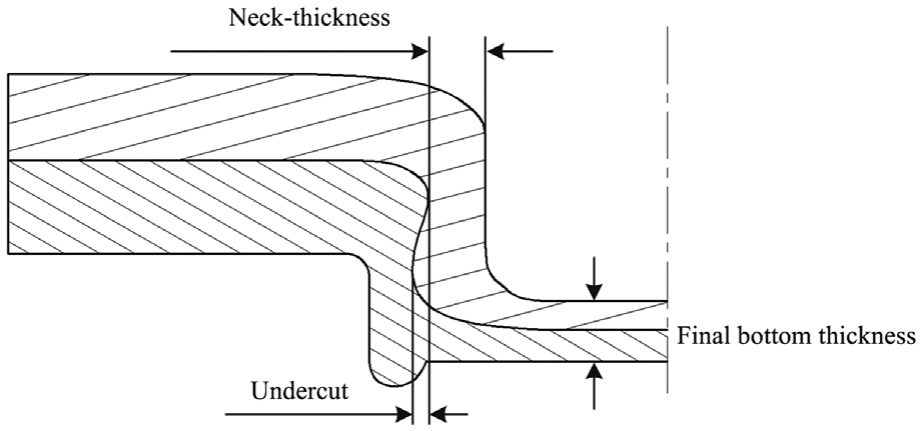
Quality assessment criteria of clinched joint.

**Figure 6.**
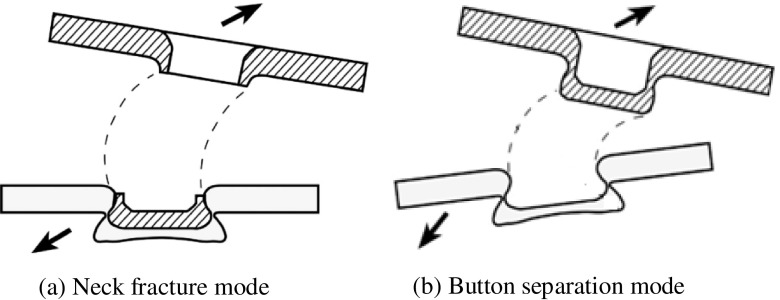
Failure modes of clinched joints [63].

The principal clinching tools include the blank holder, punch and die. The final geometry of the clinched joints is determined by the geometry of the punch and die. The final geometry in turn determines the strength of the clinched joint. As shown in Figure 5 [10], the upper sheet undergoes a significant thinning near the punch corner radius during the clinching process. The mechanical behavior of a clinched joint depends particularly on the neck thickness, the undercut and final bottom thickness. Tool design normally involves complex theoretical and experimental processes that are costly and time-consuming. The clinched joint failure modes are neck fracture and button separation [63] (see Figure 6). Small neck thickness leads to neck fracture and small undercut result in button separation. A good method design is to start with numerical simulations and validate the results by experimental tests.

**Figure 7.**
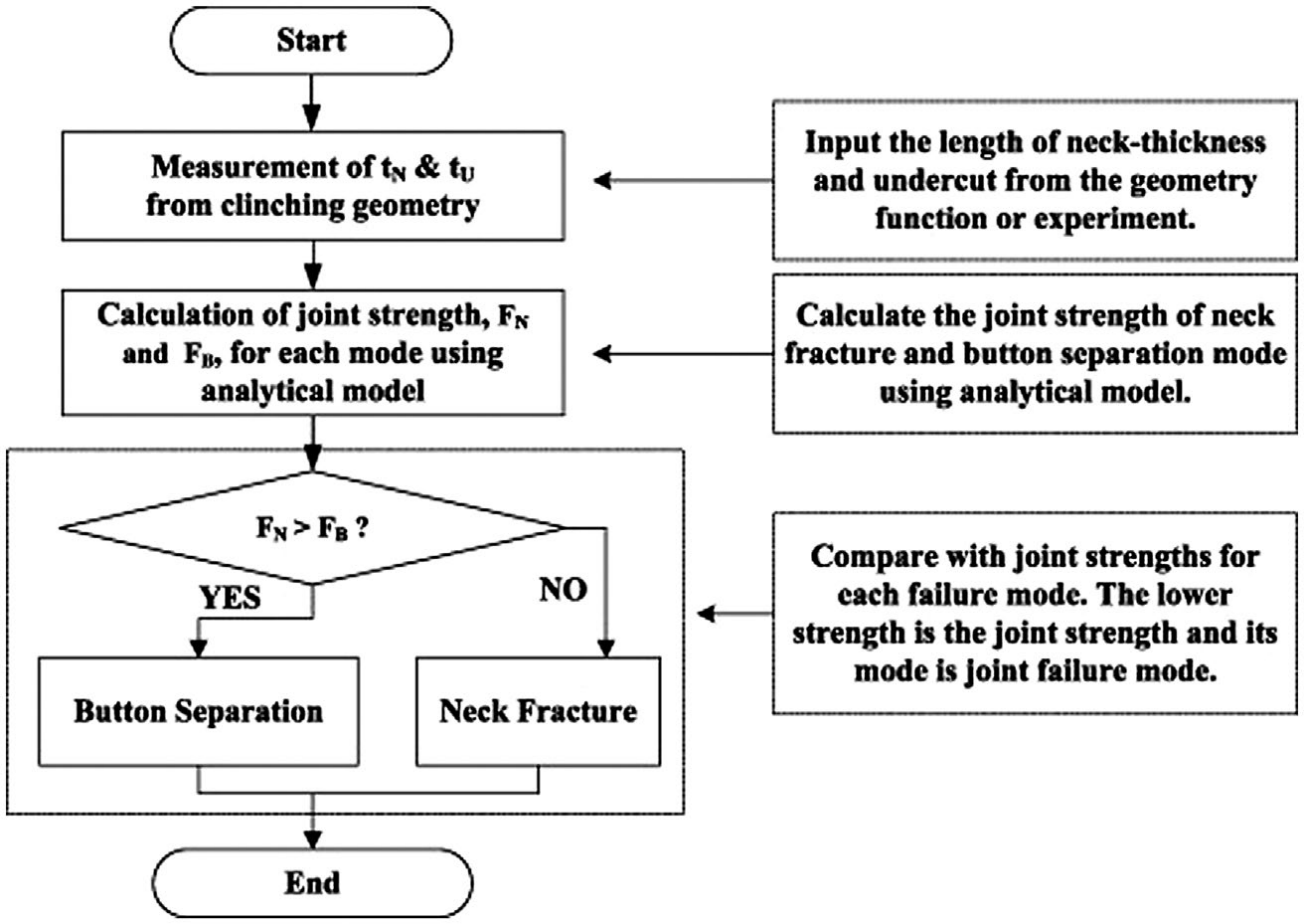
Clinching tools design procedure [66].

A clinching tools design method was proposed by Lee et al. [66]. The method is based on the analytic model, which was defined as a function of the neck thickness and the magnitude of the produced undercut. The required neck thickness and undercut were calculated from the analytic model for obtaining the desired joint strength. Figure 7 shows the clinching tools design procedure, where *F*_*N*_ is neck fracture load and *F*_*B*_ is button separation load. To optimize the conditions obtained from the proposed design method, finite element (FE) analysis and clinching tests were performed. Satisfactory agreement was found between simulated and experimental results. For evaluating mechanical properties of the clinched joint designed by this method, the H-type tensile tests for clinched joint were carried out. The results showed that the fracture load satisfied the required joint strength.

**Figure 8.**
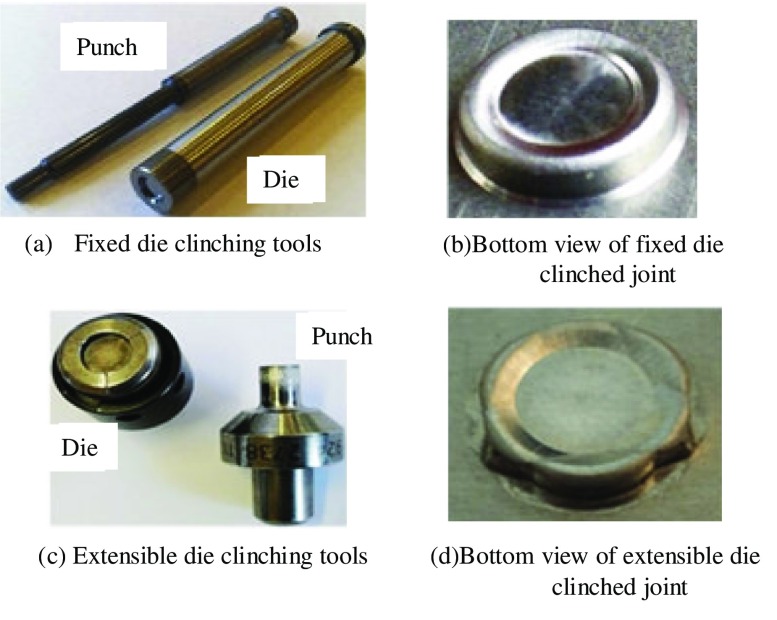
Comparison of tools and bottom views between fixed die clinching and extensible die clinching.

**Figure 9.**
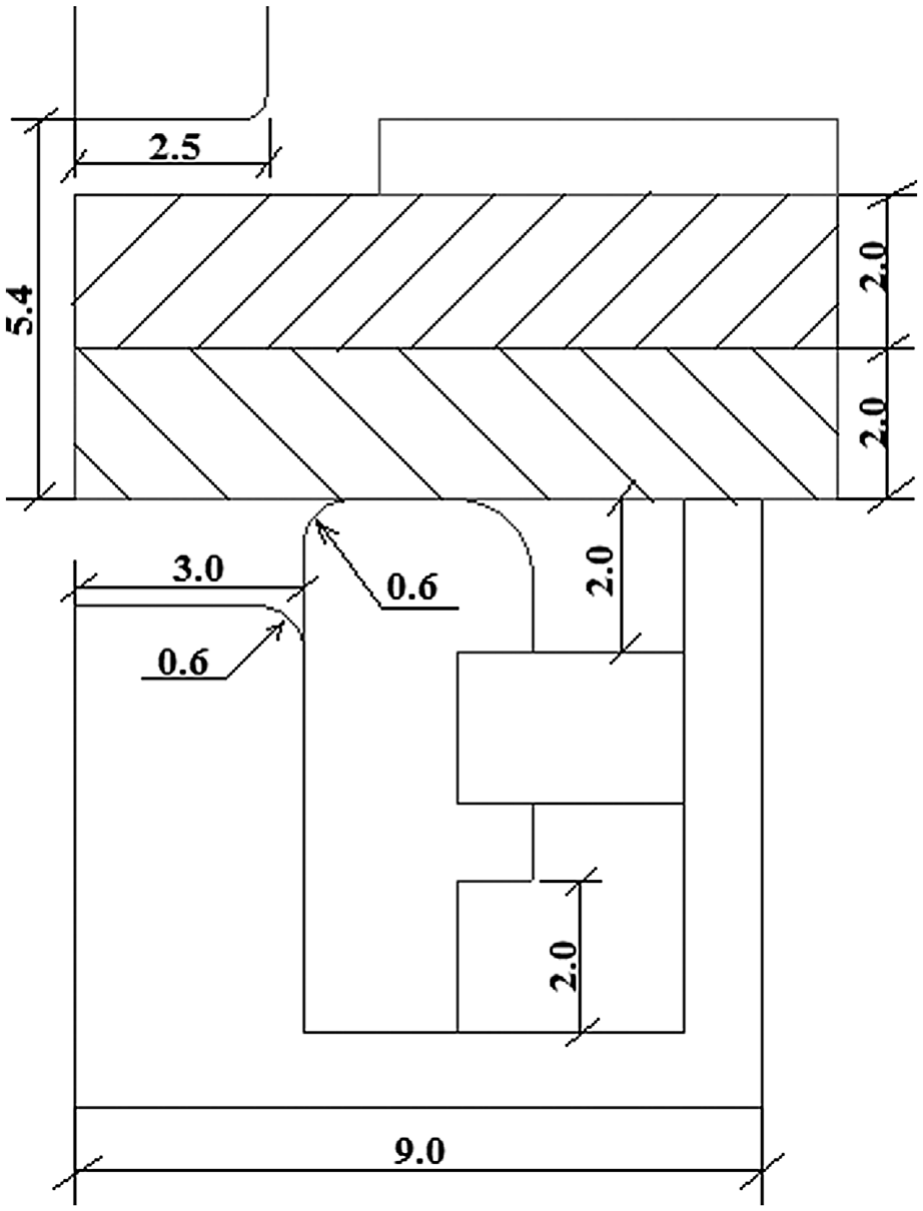
Geometrical dimensions (in mm) of a typical extensible die clinching process [62].

Another clinching configuration was developed involving an extensible die to improve the mechanical behavior of clinched joints. Figure 8 shows a comparison of tools and bottom views between two clinching configurations [62]. The use of extensible die clinching has increased in recent years. The extensible die clinching process and the mechanical behavior of the extensible die clinched joints have been studied by many researchers [62,67,68]. Figure 9 shows the geometrical dimensions of a typical extensible die clinching process. The sheet material thickness is 2 mm, punch radius is 2.5 mm, die radius is 9 mm and die groove radius is 3 mm separately [62].

Oudjene and colleagues [69–72] systematically studied the effects of tool geometry on joint performance, using the Taguchi method. An optimization procedure using the response surface methodology was presented to increase the strength of clinched joints. A response surface methodology, based on kriging approximation and adaptive moving region of interest, was presented for shape optimization of clinching tools. To avoid a local optimum and to obtain an accurate solution at low cost, the cost function was defined in terms of the maximum value of the tensile force computed during the simulation of sheet separation. Based on moving least-square approximation, another response surface methodology was also presented for optimization design of clinching tools [73]. An automatic optimization procedure was implemented in ABAQUS FE code. The results showed clearly the potential interest of the developed optimization procedure for the design of tools towards the improvement of the clinch joints resistance.

Extensible die clinching tools have also been studied by Lambiase and colleagues [74–76]. A FE model was developed to predict the effects of the tool geometry on clinching parameters. An artificial neural network (ANN) model has been utilized for predicting the properties of clinched joints produced with a given clinching tool configuration. For demonstrating the effectiveness of the proposed approach an optimization tool based on a genetic algorithm tool was developed. For evaluating the evolution of the joints’ profile experimentally, the clinched joints were produced under different forming loads. The effects of the processing conditions on the main mechanical response of the joints, namely the maximum strength, stiffness and absorbed energy, were investigated. The joints exhibited a higher strength (up to 40%) when loaded during the peeling test because of a larger interlock. In addition, the employment of extensible dies allows a drastic reduction of the forming loads, compared to those required by adopting the fixed dies.

The effect of tool geometrical parameters on the clinched joint lock size has been investigated [77,78]. It was found that the die groove width affects the material flow significantly. The results also showed the die geometrical parameters affect the joinability of advanced high-strength steel. The effects of stamping pressure and die geometrical parameters on the mechanical properties and material flow of clinched joints were comprehensively analyzed based on experiments and numerical simulations [79]. The results showed that increasing stamping pressure enhances the joint strength somewhat, and the die cavity depth influences it even more significantly.

A clinching process of sheets using a step punch has been developed to improve the joining range and joint strength [80]. In this process, the wall thickness of the upper sheet in the side wall of the punch was increased by compression with the step punch. For the joining of the thin steel and aluminum alloy sheets, the fracture in the upper steel sheet was prevented by the compression, and thus the joining range was expanded. The high strength steel and aluminum alloy sheets were joined by optimized step punch and die. The maximum static load and fatigue limit of the joint for the clinching using the step punch were increased by 21% and 78%, respectively.

Some sheet materials tend to fracture due to the weak ductility when the sheets undergo plastic deformation during the clinching processes. Several methods have been tried to avoid the sheet fracture. The die geometrical parameters were modified to relieve the deformation concentration of the sheets [81]. Using counter pressure of a rubber disk, a clinching process has been proposed to join the ultra-high strength steel sheets with low ductility [82]. In another similar study [83], a cemented carbide punch was employed to prevent the failure for a large joining force.

Geometric parameters of the clinching tool have been optimized using the methods of experimental design, statistical analysis, meta-modeling of response surface method (RSM) and genetic algorithm (GA) [84,85]. The goal was to maximize both the neck thickness and undercut value of the clinched joint. The solution set for Pareto front was obtained, which can provide engineers with a wide range of possible options. Based on process variable relationships and FE technology a fast tool design method was proposed [86]. The tensile tests and shear tests were carried out to validate the method. The results indicated that this design method could meet the requirements of the joint resistance and would greatly facilitate the design of clinching tools.

Plancak et al. [87] reported a process for joining two bimetallic axisymmetric components from various materials by upsetting the bimetallic components in a closed die. The external component was a ring and the central part was a cylindrical element. By upsetting those two elements in a closed die, an inseparable workpiece was obtained. Two different geometries were investigated. In the first case the simple ring and profiled inner cylinder were combined while in the second case the reverse combination was used. Also, the forming load, material flow and joint section were analyzed. The experimental results showed that the initial cavity was filled mainly by the outer ring material. The load exhibits gradual rise without inflexion in the curve. This investigation has yielded considerable insight into the process of joining two bimetallic components.

An air-hydraulic intensifier clinching device has been designed in which the displacement sensor and the pressure sensor were incorporated in the control loop so as to accurately control the quality of the clinching [88]. A clinching test method was found, based on experiments using two different control feedback loops with two different ways of clinching. Joint strength was analyzed and the results showed that the shear strength and fracture mechanisms for the clinched samples differ with forces applied in different directions. A clinching technology using an AC servo direct-drive device has been developed [89]. Experimental results showed that the control systems could accurately manage the connection of aluminum alloy 6061 sheets with the round joint. The joint quality was evaluated by tensile tests. The results showed that the fracture load satisfied the required strength. An added benefit was that use of the device could ensure efficiency and quality of the clinching process.

Currently, clinching is mainly restricted to joining thin sheet metal and the extended use of clinching technology to include joining thick sheet metal would be a major advance. So far, the suitability for joining substantially greater thicknesses of sheet materials has not been investigated sufficiently and tool manufacturers have not yet made available toolsets that are adapted for thicker sheets. The current state-of-the-art method for assessing the suitability of tools for joining larger sheet metal thicknesses is trial and error. This means accepting the high costs involved in producing a large number of tools and conducting empirical experiments. In an attempt to overcome this problem, Israel et al. [90,91] investigated some analytical approaches and reported their suitability for describing the process of clinching compared with the potential for calculation with FE analysis.

Varis [36,37] investigated the suitability of clinching for joining high-strength structural steel and compared the results of using round and square clinching tools. He also characterized problems encountered in the long-term use of clinching, including the lack of a systematic approach to maintenance and the need for continuous follow-up [38,39]. In a comparison of the economics of joining with and without additional elements, Varis [38] demonstrated significant differences based on established costing principles. In the case of clinching without additional elements such as rivets, the unit and total costs decrease as the tool life increases. Several points of view were discussed and calculations for factors such as marginal costs were presented [40].

## 3. Clinching process

Reliably predicting the dimensions and mechanical properties of clinched joints depends on having accurate knowledge about the clinching process itself. However, the clinching process is complex and gaining insights into the process of joint formation is difficult and time-consuming. Experiments and numerical simulations help overcome the barrier by offering an effective way of studying the formation of clinched joints [92–94].

### 3.1. Clinching process parameters

The characteristics of clinched joints can be influenced by changing the process parameters. These must be selected, therefore, so that resulting joints are fit for purpose. Crucial in this respect is the formation of the neck and undercut which is influenced by many factors with multi-factorial interactions. Much experimental and numerical research has been carried out with the aim of quantifying these factors and optimizing the parameters influencing the clinching process [95–97]. A systematic study on the mechanism of clinching was reported by Budde and colleagues [98–104]. The authors defined some basic terms, such as mechanical contact chains joint networks for establishing a basic theory for studying the mechanism of clinch-joining.

Quality control systems for clinching were developed during the 1990s. A real-time automated system was reported by Bober and Liebig [105] and Liebig et al. [106, 107]. An online quality control system was reported by Eckold in 1994 [13]. The monitoring systems can distinguish between accidental and systematic process errors and can therefore minimize plant stoppages and ensure high levels of plant availability [108]. For example, the VISICON IST Research and Development project has developed a distributed real-time quality control system covering the production of clinch-joined galvanized metal boards. The system uses modern computing concepts including an image processing component based on the G Transform. The solution has been validated by statistical analysis of a large dataset covering the detection of joint buttons and assessment of their quality [109, 110].

A window monitoring system can be used to study and evaluate the quality of clinched joints. Tan et al. [111] investigated monitoring functions for combinations of clinched materials, sheet metal thicknesses and clinching machine tools. The results showed that the method offers an outstanding capability for monitoring changes in joining conditions, thereby assuring reproducible quality in the joints. The window monitoring method has two main advantages over the conventional tolerance monitoring method. Firstly, the monitoring phase is shorter, and secondly, more information is made available. The additional information makes it easier to distinguish which errors occur during a clinch joining operation.

The large joining forces requiring heavy process equipment are a major impediment to the use of clinching. In response to this problem, the Fraunhofer Institute (Stuttgart, Germany) has developed new clinching methods that involve much lower joining forces. These developments could make it possible to use clinching as a joining method for a range of new application areas [112].

Where different materials need to be joined by clinching, a different set of problems must be solved. Lee et al. [113,114] used a combination of FE analysis and laboratory tests to study the effects of varying the process parameters on the characteristics of clinched joints between sheets of high-strength steel DP780 and Al5052 alloy. Differences in ductility between the two materials being joined were found to be the main contributing factors leading to joint defects such as necking of the upper sheet, cracks in the lower sheet, and a failure to form an adequate interlock. The results suggested that the joinability of combinations of these materials was affected mainly by the die radius, die depth, and the shape of the die groove. Joint strength was found to be determined by the interlock length and neck thickness.

Numerical simulation can be used to predict the locking strength of clinched joints and provide guidance on the design of the forming process. For example, Wen et al. [115] carried out simulations and experimental tests for understanding the effect of varying parameters on the clinching and stripping processes for joints between aluminum AL6016 and H70 brass sheets. The authors found that with increasing punch radius, the neck thickness increases and the undercut decreases. They also found that increasing the depth of the die groove causes a decrease in the neck thickness and an increase in the undercut. The authors recommended that the bottom thickness should be 25% of the total sheet thickness.

The mechanical strength of a clinched joint can be optimized by employing a global optimization technique based on a Kriging meta-model. For example, Roux et al. [116] reported a process that included optimization of the joining stage and simulation of the mechanical strength of the resulting clinch joints. The authors showed that it is essential to account for plastic strain, residual stresses and damage when studying the mechanical strength of clinched joints. The authors showed that using the global optimization procedure enabled the achievement of a 13.5% increase in the mechanical strength of clinched joints under tensile loading and up to a 46.5% increase for shear loading.

Clinching does not involve applying heat to make a joint and this makes it possible in some situations to join pre-coated materials. However, if clinching is to be used as a joining method in the visible parts of products then the damage to the coating caused in the process must be minimized. Accomplishing this involves optimizing the process taking into account several factors including the type of sheet metal, the coating material, the geometry and material of the tools, the forming temperature and the lubrication condition of the sheet metal surfaces and the tool surfaces. Behrens et al. [117] investigated ways of reducing the damage to the coating when clinching aluminum sheet metal. Heat treatable Al–Mg–Si alloy Ac-170 with a sheet thickness of 1.0 mm was chosen because it is commonly used in the automotive industry for outer hang-on panels. The research aimed to optimize a range of process factors including the macro-geometry of the clinching tools, the choice of tool materials such as steel and ceramic, the effect of the micro-structure of ceramic tools, the impact of different coatings and the influence of micro lubrication. The authors presented results comparing ceramic and steel tools, different coatings, and mechanical properties of the clinching elements. The results showed that with suitable lubrication conditions, the steel clinching tools satisfied the required optimum conditions for minimizing the damage to the coating when clinching aluminum sheet metal.

Research on the application of clinch joining methods is not confined to transportation. LaBoube [118] considered issues related to the performance of an open web I-joist for use in building construction. The authors investigated the configuration of clinch joints needed to manufacture a closed chord profile beam with sufficient shear strength and capacity to withstand flexure. The authors found that the distance between clinch joints should be no more than three inches if premature failure of the joints under load is to be avoided.

### 3.2. Process modeling techniques

Finding equations for predicting the mechanical properties of clinched joints is made difficult by their complex three-dimensional geometry. Equations for predicting the mechanical property of one or more clinched joints have been generated for limited cases [119–121]. However, determining the mechanical property of joints by calculation appears to be case-specific. FE analysis and neural networks are now used commonly to study clinching processes [61,122,123]. The FE modeling is a mature and essential part of many clinched joint design practices that have led to improved designs and reduced development times and costs.

In general, two methods, the ‘implicit’ and ‘explicit’ methods, can be used in FE modeling of clinch forming. The implicit method is more common and used in a wide range of FE codes for solving linear and nonlinear problems [63]. The explicit algorithm is suitable for analyzing dynamic problems particularly where contact is important [124]. Because the clinch joint dimensions are of the same order of magnitude as sheet thicknesses, simulation must be conducted for the bulk metal forming process [74]. Sheet material modeling is very important and complicated in the clinching process. Most researchers use the von Mises yield criterion. Some researchers use multi-layer compression tests and others use methods such as the one proposed by Coppieters and Kuwabara [125] to retrieve the strain hardening curve for large plastic strains.

Information about clinching processes that can be obtained from FE simulations includes the flow of the materials being joined, the die fill, the strain distribution, rates of strain in the materials, the distribution of pressure at the interface between the material and the die and the influence on the joint formation of properties such as friction. Solving these problems involves confronting issues such as the plasticity of the materials, large deformations in the materials and interactions at the point of contact. Numerical simulation based on dynamic or static implicit and explicit methods can be used to help solve such problems and industrial software (e.g. ABAQUS, ADINA, LS-DYNA 3D and MARC) already enables this to be done [126–128].

FE simulation has the great advantage that the mechanical properties of almost any shaped clinch joint can be determined under various load conditions. These computerized methods have been used to determine the most appropriate tools for particular combinations of materials and to design alternative tools. The results of simulations have also been used to improve the robustness of clinching processes and process monitoring as part of quality assurance measures. In early research work related to FE analysis, Hahn et al. [129,130] presented joining-spot models which can be used to simulate sheet material joints using FE methods.

**Figure 10.**
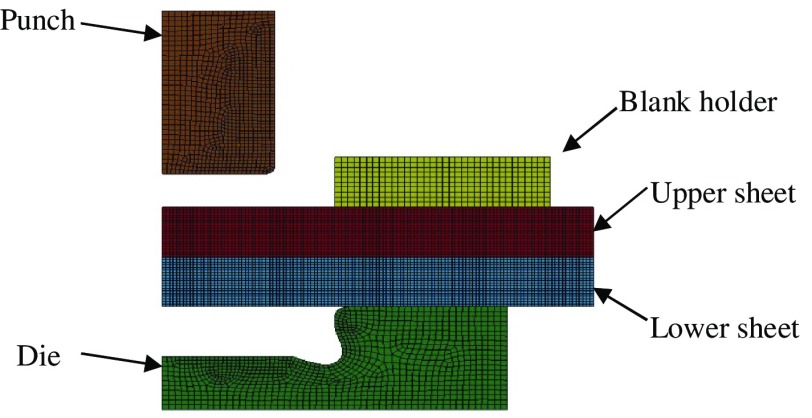
FE model of the clinching process [63].

**Figure 11.**
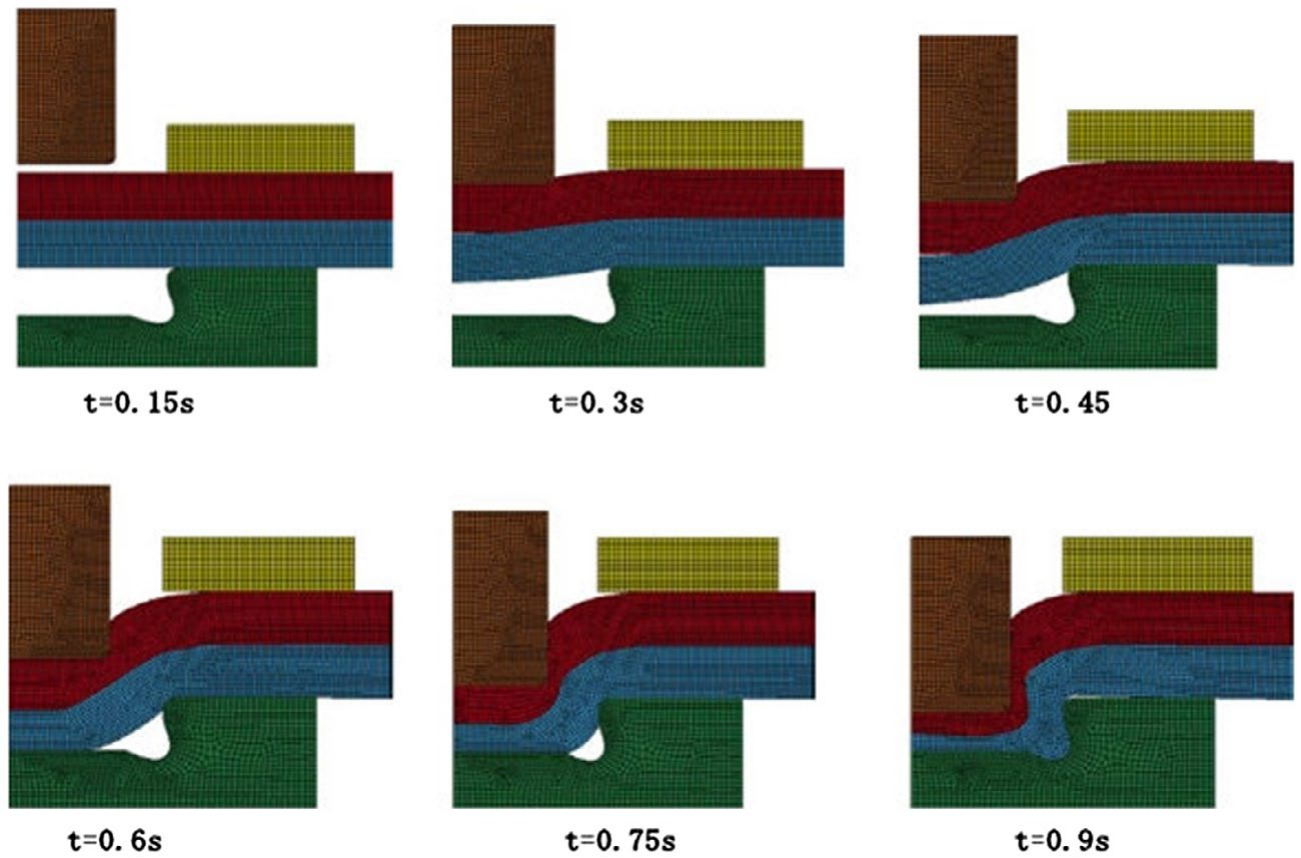
FE simulation of the clinching process [63].

He et al. [63], Feng et al. [131], and Yang et al. [132] have used the commercially available LS-DYNA software to generate axisymmetric FE models based on the Cowper–Symonds material models (see Figure 10). The implicit method using a Lagrange method and *r*-self-adaptivity was employed and the simulation is illustrated in Figure 11. Simulations of the clinching process were validated by conducting tests on specimens made of aluminum alloy 7075.

**Figure 12.**
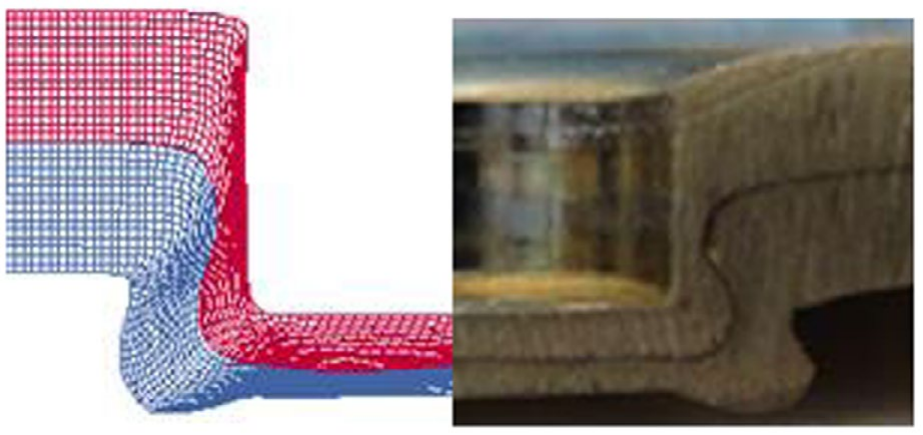
Cross-section comparison [62].

A comparison between numerically simulated and actual cross-sections of clinched joints showed reasonable agreement between the two, as shown in Figure 12 [62]. The neck thickness, the undercut and the final bottom thickness of the actual clinched joint are 0.35, 0.29 and 1.41 mm separately while that of the simulated clinched joint are 0.33, 0.34 and 1.39 mm separately. There are small differences between the actual and simulated clinched joints and this is probably because the material transverse anisotropy was not accounted for in the simulations. Anisotropy is the material property of being directionally dependent and transverse anisotropy is associated with the tensile strength of sheet materials. The measurement of the transverse anisotropy constants of the lightweight sheet materials is still a difficult task.

**Figure 13.**
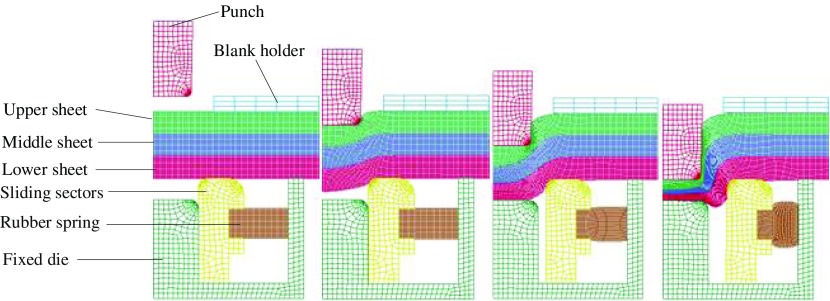
FE simulation of the three layers clinching process [133].

Some practical situations require more than two components to be joined by clinching. In order to further understand the process, Yang et al. [133] investigated the formation and characteristics of clinched joints comprising three layers with different materials and thicknesses. Three FE models were used to simulate the extensible die clinching process for three different specimen configurations. The FE simulation for a three layer clinching process is shown in Figure 13.

Cheong et al. [134] developed a rigid-plastic FE method using Eulerian meshing to deal with the problem of large deformations during metal forming. The complicated re-meshing in the Lagrangian approach was replaced by a system in which the initial elements were fixed in a specified region with particles implanted as markers. Comparisons between the material flows, punch loads versus stroke and the dimensions of the final products, obtained from simulation runs and experiments, showed that the proposed simulation method effectively represented real-world experience.

A FE procedure incorporating automatic re-meshing was developed by Hamel et al. [124,135,136] specifically to simulate the process of clinching. In this procedure a static explicit approach was used to resolve the updated Lagrangian formulation. A Simo and Taylor algorithm was used to realize the integration of the elastic-plastic behavior law, and a penalty method was employed to ensure the contact conditions. The results of simulations using the procedure were compared with experimental data. Sahyoun et al. [137] used an adaptive re-meshing procedure to derive an accurate numerical evolution of the final geometry. The procedure included error indicators and field variable transfer built by a meshless technique known as diffuse approximation. The numerical results from simulations agreed well with experimental data. A procedure for selecting an appropriate combination of clinching tools was proposed by Varis and Lepistö [39]. The calculations included the resulting bottom thickness, X, produced by several tool combinations. The simulations demonstrated the easy contact definition of the MARC software. A survey of the approaches available in ABAQUS has been presented [138]. Various applications of these techniques were discussed through a series of case studies. The results showed that though the modeling techniques were motivated by automotive applications, they can also be used in other areas such as aerospace structures.

Back propagation (BP) neural networks can be used to map the relationship between the joining parameters and the mechanical properties of joints between steel and aluminum used in car bodies [139–141]. To overcome the lower training precision, low convergence speed and weak generalization capability of the standard propagation algorithm, the Levenberg–Marquardt and normalization algorithms were combined to optimize the standard BP neural network connection weights and improve the model prediction precision and generalization capability. Training and validating samples were performed using the BTM^®^Tog-L-Loc system with different combinations of parameter values. Then the parameter values for the training samples and the corresponding joint mechanical properties were supplied to the neural networks for training. Finally, the experimental data were used to check the predictions.

Numerical simulation and experimental research was carried out by Huang et al. [142] to study the feasibility of clinching 1.5 mm thick steel and aluminum sheets. The validity of the simulation model was verified by experiment. The connecting features of steel–aluminum sheets with different strength grades were investigated. The authors found that to obtain high-quality joints as judged by shear and tensile strength, the sheet with the lower yield strength should be the upper sheet, and the yield strength of the two sheets should be as close as possible to one another. Optimal solution sets were obtained by matching the material type and sheet thickness of steel-aluminum sheets through multi-objective optimization using approximation models for the neck thickness and the undercut of the joints.

Material damage during the clinch joining process has been studied using the Gurson–Tvergaard–Needleman (GTN) model [143, 144]. The authors first identified the parameters of the GTN model which influence the void nucleation, growth and coalescence in the materials being joined. The parameters were then imported into a FE simulation model. The results of simulations demonstrated that incorporating concepts from the GTN damage model can extend the predictive capability of FE simulation models. Ippolito et al. [145] carried out numerical simulations and experimental investigations according to a factorial experimental design in order to build a knowledge base of tool shapes and the corresponding mechanical and geometric characteristics of clinched joints formed by using them. Optimal values for the variables considered were found by a regression analysis. Experimental evidence confirmed the validity of their approach. The potential use of FE-based methods for conducting sensitivity analysis and optimization studies for clinch joining was presented by Drossel et al. [146]. The authors derived the most important values for forming die optimization by determining the sensitivity of the design to variations in the connection parameters. The robustness of the clinching process in production was evaluated by conducting a sensitivity analysis of the uncertain variables.

The Coulomb friction and constant shear friction models can be used in elastic-plastic and rigid-plastic FE analysis to investigate the behavior of the clinching process. Jayasekara et al. [147] chose to investigate the consequences of varying die depth, die diameter, groove corner radius, and groove width on joint characteristics. The strength of clinched joints was evaluated by examining the separation strengths, such as peel strength and tensile shear strength. A failure diagram was constructed that summarized the analysis results. Simulation results showed that the die diameter and depth were the most decisive parameters for controlling the quality of the clinched joints, while the bottom’s thickness was crucial for determining the separation strengths. Yang et al. [148] developed an ANSYS FE model based on elastic-plastic FE theory and used it to investigate the effects of varying the die parameters and sheet thickness on the resulting neck thickness and undercut of clinched joints.

Studies have shown that the SPR2 model can be used in place of more complex models to study clinching processes. For example, beam elements, solid elements and constrained elements (SPR2) models were used in to simulate quasi-static, cross-tension and lap-shear tests for joints between aluminum and steel [149]. SPR2 simulations showed a lower value for the cross-tension force than was obtained in physical tests but otherwise corresponded well. Solid element and SPR2 models can be used instead of beam element models to simulate ductile failure in lap-shear. FE simulations were also carried out for clinching processes of steel–aluminum material combinations [150].

For obtaining optimal parameters combination subjected to multiple characteristics, Eshtayeh and Hrairi proposed a method of Taguchi-based Grey optimization in clinching [151,152]. LS-DYNA software was employed for evaluating the joint strength. Taguchi’s L27 orthogonal array design and the notion of signal-to-noise (S/N) ratio were utilized to obtain the objective function. The results provided the optimal geometrical parameters of clinching tools.

Analysis of the clinch forming operation using FE-based methods requires data on flow stress and values for the friction at the tool–sheet and sheet–sheet interfaces. Because the forming process involves severe local plastic deformation of the materials being joined, the stress–strain curves obtained from standard tensile tests have to be extrapolated according to a hardening law. The extrapolation method used strongly determines the error on the load-displacement curve obtained by numerical simulation. Also, the friction at the interface affects the flow of material and this determines the final interlock and the strength of the joint. Coppieters et al. [153] reported a punch compression test for sheet metal using circular specimens and an optimization strategy based on gradient. The test enables the interface friction and material behavior to be identified simultaneously. The optimal geometry for a specific application has also been investigated with FE simulations [41, 154]. The authors demonstrated the difficulties that can be encountered in using FE simulation and discussed ways in which they can be overcome so that the physical strength of new types of connections can be predicted reliably. It is difficult to involve local deformation sub models in FE analysis of complicated clinching process. Breda et al. [155] use a simplified element method to represent the equivalent modeling strategy for FE simulation of large structures. The key point of the method is the use of uncoupled plastic behavior to model the joint plastic properties. For calibrating the parameters governing the equivalent model, a simple shear lap and pull-out reference test of a single clinched joint was used. The presented methodology was validated using a modified Arcan test of a single joint, which allowed to exert a combination of shear and pull-out loads.

Traditional manufacturing of vehicle catalytic converters employs two types of bending die for combining upper and lower heat insulation plates. A single-action, two-step clinching system for joining the plates has been developed in order to simplify the process [156]. The authors used FE analysis in the design stage for predicting bending force and plate final shape. Then a bending die recovery and cushioning system can be designed. A study involving a combination of experimental trials and numerical simulations of the clinching process was carried out and the mechanical strength of the product with different surface preparations was ascertained [157]. FE simulations with ABAQUS/Explicit software were used to model the clinching process and the performance of the joints under shear and cross tension loads. The punch force-displacement curve and the observed strength of the clinched joints were investigated to understand the influence of the plastic anisotropy of the material under load.

The current strategy for lightweight body in white design in the automotive industry is to use a mix of materials. In this context, Busse et al. [158] investigated the clinch joining of AA6014 and press-hardened 22MnB5. Simulations using FE methods were analyzed to verify different tool concepts and to establish appropriate values for process parameters. Physical experiments were then conducted to demonstrate the feasibility of the clinching process. Quasi-static cross tensile strengths and shear tensile strengths of different clinching parameters completed the experimental investigations.

Technology has been developed recently that enables unlimited sheets of the same or different metals to be joined continuously in a process known as roller clinching. The main problems encountered so far have been asymmetry of the resulting clinch points and fact that the impact of the punch and its retraction are not perpendicular to the sheets being joined. In order to understand the material flow during the formation of roller-clinched joints, Rill et al. [159] developed a FE simulation model and used it to study the influence of various parameters upon the material flow. The results of simulation runs were validated by comparison with results from laboratory experiments. The authors presented an approach to optimization based on the insights obtained from the simulations and practical experiments. A FE analysis based on the ABAQUS software was also carried out for the C-frame of the roller clinching machine [160]. Variations of frame geometry were assessed by analyzing the straining of the material. Some of these variations included reduction of the mass of the frame by recessing in order to determine the influence upon the rigidity of the structure.

Life cycle assessment (LCA) is used in the design and development phases in order to optimize the economic and environmental impacts of products from concept to disposal. When considering the manufacturing phase of the product life cycle, it is usually necessary to generate models of individual steps and then analyze them in combination. For this modeling to be successful it is necessary to develop parameterized processes [161] so that the effect of variations in the parameter values can be properly assessed. The aim of the modeling effort should be to provide designers with tools that include parameterized models of environmental processes that are applicable to the products being considered. With such tools, designers would be better able to estimate important elements such as the probable energy consumption and materials for the manufacturing technology used.

## 4. Clinching of different sheet materials

The vehicle manufacturing industry envisions that optimization of vehicle cost and performance can be achieved by using a range of materials for vehicle parts and assemblies so as to take advantage of the particular properties of each material. Following this philosophy, some automobile companies are experimenting with lightweight materials to improve energy efficiency and performance while simultaneously reducing carbon dioxide emissions. A recent lightweight concept vehicle was constructed using ultra-high-strength steels, carbon-fiber composites, aluminum, magnesium, and advanced lightweight plastics throughout. The range of materials with substantially different properties raised particular problems with respect to joining. In this context, clinching is often a preferred joining technique for aluminum alloys and steel as well as for metals such as magnesium alloys, titanium alloys and composites that are difficult to weld [162,163].

### 4.1. Magnesium alloys

The availability of lightweight magnesium alloys in recent years has provided further opportunities for weight reduction in transportation system components. A pre-condition for the expanded use of these materials is the availability of ways to join components that are widely applicable and offer maximum utilization of the materials under operating loads [164]. Clinching can be used to join magnesium components homogeneously as well as in combination with other materials. Unfortunately, plastic deformation of magnesium and its alloys can be achieved only at temperatures above about 220°C because of the anisotropic deformation of the hexagonal crystal structure at lower temperatures. Heating magnesium substrates above 220°C leads to a broad extension of deformability and enables the formation of high-quality clinched joints. Hahn et al. [165, 166] described a laboratory-scale process for safe joining of magnesium substrates by clinch riveting, self-piercing riveting and clinching. This was achieved by inductively heating the magnesium substrates as a precursor to the joining operation.

The flat clinching method also offers important benefits for joining magnesium [167–169]. Flat clinching employs a flat anvil in place of a die. When forming Mg/Mg, Al/Mg or Fe/Mg joints with a flat counter tool, the proportion of crack inducing tensile stresses in the bottom part is very low. Shi et al. [170] studied the effect of elevated temperature on the quality of clinch joints between sheets of AZ31 magnesium alloy. The authors simulated the transfer of heat energy during the clinching process using commercially available DEFORM-2D and DEFORM-3D software. Simulations and subsequent analysis covered different values for the groove fillet radius, die depth, groove depth, groove width and draft angle [171, 172]. Results from studies of the main geometric parameters of joints between magnesium alloy samples showed that joint strength can be effectively improved by suitably controlling the die parameters.

### 4.2. Titanium alloys

**Figure 14.**
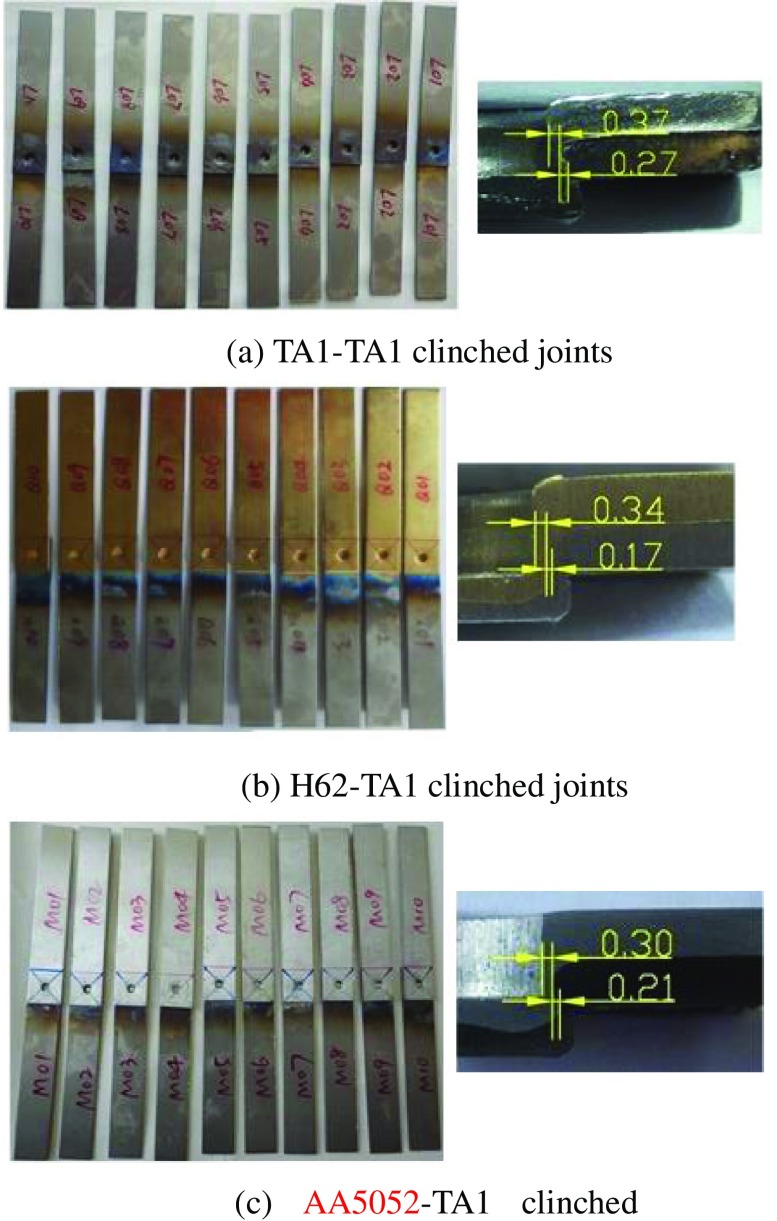
Clinched joints between similar and dissimilar metal sheets (dimensions are in mm) [174].

Titanium alloys have also been used in place of steel in order to reduce vehicle weight and lower fuel consumption. Fusion welding is commonly used to join titanium sheets together or to join titanium to other metals but this process can change the micro-structure of the welded region, adversely affecting the mechanical properties of the joints. In addition, fusion welding requires materials and equipment and the means to discharge the exhaust gases. These requirements increase manufacturing costs and impacts on the environment. It is widely accepted that clinching can be used to join titanium and other lightweight alloy sheets well but few investigations have been published to date. He et al. [173,174] reported experiments on the mechanical behavior of titanium clinched joints. Joints between combinations of similar sheets (TA1–TA1, where TA1 is an industrial code for titanium metal with less than 1% of impurities) and dissimilar sheets (H62–TA1 and Al5052–TA1, Figure 14) were studied. The tensile-shear strengths of the joints were characterized by plotting force–displacement curves and the energy absorption, load-bearing capacity, and failure modes were also studied. Results showed that almost all titanium sheets clinched joints failed in the neck fracture mode. Results also showed that the load-bearing capacity and energy absorption of clinched joints with titanium as upper sheets are higher than those of the clinched joints with titanium as lower sheets.

### 4.3. Copper alloys

Copper alloys have excellent ductility, thermal and electrical conductivity. Because of these properties they have been used extensively in different engineering fields. Combinations of dissimilar metals such as copper and aluminum alloys have also been widely used in electrical industries. Where similar and dissimilar metal sheets of copper alloys and aluminum alloys must be joined, fusion welding and friction stir welding techniques are normally used. It is widely accepted now that these materials can also be joined by clinching because copper and aluminum alloys have excellent ductility. Several authors [64,175,176] have reported clinching of the H62 copper alloy. The authors conducted tests on mechanical properties of joints formed by clinching. Experimental data were validated by statistical analysis. Results for fatigue life (N) and fatigue load (F) were plotted on log-log coordinates and fitted by a least squares method. Equations describing the F-N curves were obtained and analysis of the results showed that the clinched joints in sheets of the copper alloy exhibited favorable fatigue performance.

### 4.4. Composites

Composites are increasingly being incorporated into lightweight, multi-material designs. This is especially in car industries. For achieving an optimal mechanical property of these hybrid structures, it is important to use appropriate joining methods [177–179].

Gude et al. [180] reported the adaptation of classic clinching for thermoplastic composites to manufacture hybrid structures incorporating continuous fiber-reinforced thermoplastic and metallic components. The process was based on thermo-clinching and the resulting joints were subjected to destructive and non-destructive testing under laboratory and manufacturing conditions. Joining metals to plastics is usually accomplished by using mechanical fasteners such as bolts or rivets. This involves drilling holes, which may be expensive [181]. Investigations have been carried out on the feasibility of forming plastic-metal joints by clinching [182]. These authors reported the development of a prototype apparatus that preheated aluminum alloy AA5053 and polystyrene sheets before clinching. Tests conducted as part of the development involved varying the values of the main process parameters including the pre-heating conditions and forming pressure. The results of microscopic examination of the joints produced under different operating conditions were reported. The main failure modes were identified and the effects of variations in the values of process parameters on the mechanical behavior of the joints were clarified. The experimental results showed that the threshold in the joining force is not influenced by the preheating conditions and clinching allows the joining time to be greatly reduced.

A new concept for producing a mechanical interlock between plastics and metals was reported by Grujicic et al. [183,184]. This approach, called clinch-lock technology, incorporates some of the ideas from the spot-clinch joining process. The authors carried out FE-based analyses involving sheet-metal forming, injection molding and structural mechanics for assessing the potential of clinching in order to provide an appropriate level of interlock between metals and polymers. Results showed that levels of stiffness and resistance to buckling comparable with those observed in the competing injection over molding polymer-metal process can be attained. The new process had the additional advantages that no drilling was required and there was a 3% reduction in the weight of the polymer component.

**Figure 15.**
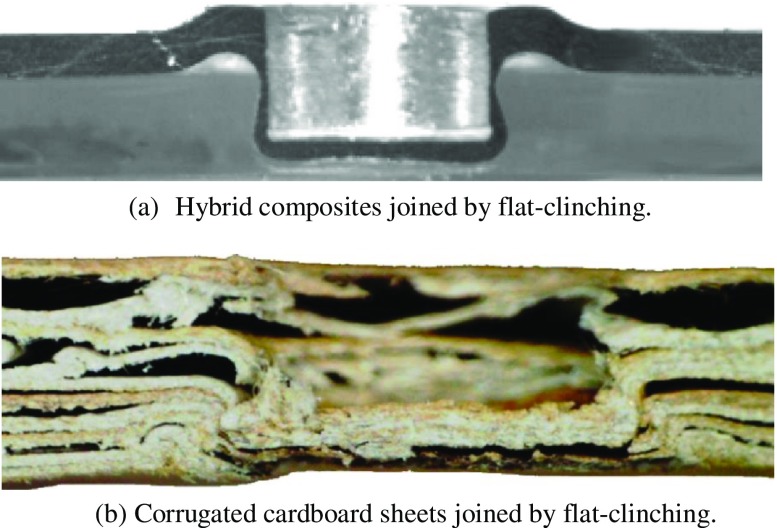
Cross sections of composite and cardboard sheets joined by flat-clinching [185].

Composite structures comprising plastic and metal components may be joined by a combination of gluing and mechanical forming. In most cases the components must be fixed in place while the adhesive cures. This increases the overall process time or adds steps to the process. These problems can be avoided by using a flat-clinching process in which plastic and metal components are joined without extending the process time, to allow for curing of the adhesive and without leaving an exterior protrusion that necessitates further finishing work [185]. A cross-section of a typical flat-clinched joint between aluminum (punch-sided) and polystyrene (anvil-sided) is shown in Figure 15(a). Sheets of cardboard are typically joined by gluing or stapling. As noted above, the addition of a gluing step in the process increases the process time while stapling involves additional joining elements (staples). However, it is possible to join certain kinds of cardboard by flat-clinching. This reduces the joining process time by eliminating the heating, application, and curing steps involved in joining by gluing and also the laborious maintenance process of cleaning the glue application equipment. Figure 15(b) shows the cross section of a flat-clinched joint between two pieces of corrugated cardboard [185].

The applicability of clinching for Al-polymer sheet combinations was studied by Lambiase and colleagues [186–188]. For assessing the influence of the punch-die cavity geometry upon joinability, different punches were used. Morphological analysis was performed to determine the main joint dimensions and the damage produced on the aluminum and carbon fiber reinforced polymer (CFRP) sheets. The effectiveness of two-step clinching for joining aluminum and CFRP was also investigated [189]. Different reshaping tools were involved after joining by clinching with split dies. The reshaping force was also varied. Single lap shear tests of simple clinched and reshaped specimens were carried out for determining the effect of the process conditions upon the performance of the joints. The morphology and geometry of joints were analyzed to understand how the reshaping step influences the main quality criteria of the joints and the damage produced on the CFRP sheet. The suitability of clinching to join glass fiber reinforced polymer (GFRP) sheets with aluminum sheets was investigated by Lambiase et al. [190]. Different types of tools were tested, including split, grooved, flat dies, as well as rectangular ones. Effect of sheet thickness and alloy composition upon joinability and mechanical behavior of the joints was analyzed. Single lap shear tests were conducted to study the joinability of the GFRP sheets with aluminum sheets. In addition, the geometry evolution of the joints during clinching was studied to understand the material flow and damage evolution of both aluminum and GFRP sheets.

## 5. Hybrid joining

Hybrid joining involves using two or more joining techniques to produce joints with desirable properties in addition to those obtained by using a single technique. The mechanical properties of clinched joints can be improved by combining clinching with other fastening techniques, such as adhesive bonding, riveting, etc [191–194]. Experimental analysis of hybrid joints can be conducted using the design of experiments (DoE) methodology, taking into account the influence of factors such as the materials being joined, tool and joint geometry and environmental factors on the static strength, stiffness and energy absorption of the resulting joints. Results can be assessed using statistical methods such as analysis of variance (ANOVA) [195].

### 5.1. Bond-clinching

Adhesive bonding is used mostly to enhance the load-bearing capacity of joints and make them watertight. Combining clinching with adhesive joining appears to have potential applications in manufacturing but there is at present some uncertainty about the stability of hybrid joining processes. Most of this uncertainty arises from a lack of knowledge about the flow of adhesive during the joining operation [196].

Sadowski et al. [197–201] conducted systematic studies involving numerical simulations and experimental tests of clinched lap joints between dissimilar metal strips bonded with adhesives, and reported results that can be applied in different engineering fields. FE models of the hybrid joints were constructed using the ABAQUS code and used to study the performance of the joints under tension and shear loads. The study aimed to investigate the whole-structure response up to and including eventual failure. Experiments using the digital image correlation (DIC) system ARAMIS allowed precise monitoring of the deformation process in a selected hybrid joint. The experimental and numerical simulation results confirmed that the introduction of an adhesive into the clinched joint increases the joint strength.

While it is commonly believed that adding an adhesive layer to clinched joints is beneficial, there is uncertainty about negative effects on the mechanical properties of the joints. Several researchers, e.g. He et al. [63], Xing et al. [202], and Liu et al. [203] have investigated the strength and energy absorption capacity of clinch-bonded joints under tensile loads in order to resolve this issue. The results of their work show that including an adhesive layer increases both the strength and energy absorption capacities of joints. They found that the adhesive layer can act as a lubricant between the two sheets during the clinching process but that after curing, the glue introduced strong adhesive forces between two sheets. However, in tensile shear load tests the adhesive layer failed in a brittle manner after peak load. At this point, the clinch keeps sheets connected but the joint can bear only a small additional load.

Friedrich et al. [204,205] investigated using hot-melt adhesive bonding in clinch joints between thermoplastic and metal sheets. Specimens of PA6-GF15 plastic and DC04 steel were clinch-joined in various combinations and then subjected to peel and tensile shear loads. The authors were able to demonstrate that appropriate clinching and hot-gluing can result greater joint strength compared to clinching or hot-gluing alone.

FE methods are commonly used to simulate the failure of clinch-bonded joints. Alternatives include using GTN and ductile damage (DD) models. Cohesive zone (CZ) models can also be used to simulate damage and failure in clinched joints that incorporate an adhesive layer. Excessive distortion of the elements can be avoided by using arbitrary Lagrangian–Eulerian (ALE) adaptive meshing and the minimum time increment in explicit analyses can be increased by using mass scaling. On this basis, Pirondi and Moroni [206] began their analysis with 3D modeling of clinching processes. They then derived the values for GTN and DD model parameters by an inverse method, comparing the results from simulations of simple clinched joints with data from experiments or published literature. Finally, the adhesive layer was introduced into the model as a layer of cohesive elements. The results of the simulations agreed well with values obtained experimentally with respect to the stiffness, peak load and the energy absorption capacity of the joints.

The purpose of Lee et al.’s study [207] was to propose multi-cohesive zone models (CZMs) for the design of hybrid joined structures made from AA6063 alloy sheets. The failure behavior of hybrid clinching was described by multi-CZMs to evaluate the crash resistance of hybrid clinched structures. Cohesion parameters of mechanically joined and adhesive bonded parts in hybrid joining were determined by a numerical-experiment approximation technique. The crash-worthiness of top-hat specimens joined by hybrid clinching was designed based on the crash analysis with multi-CZMs. Also, the crash tests were performed to verify the effectiveness of multi-CZMs.

### 5.2. Ultrasound-assisted clinching

Ultrasound-assisted clinching applies ultrasonic energy to the punch during the process. Current research focuses on the possibility of using the ultrasonic softening mechanisms in clinching of high-strength steel and aluminum. An ultrasonic unit with a power of 1 kW was installed into a C-shaped clinching bow to apply a vibration with a frequency of 20 kHz to the clinching process. Heeln and Wanner [208] described the integration of the ultrasonic unit in the clinching process. The research results focusing on verification of the concept were also presented.

### 5.3. Rivet-clinching

Mucha et al. [209–211] proposed a new sheet metal joining technology using a combination of clinching and riveting. The authors described joining S350 GD sheet metal using this combination of joining technologies. The authors carried out uniaxial shearing test to determine the strength of overlay joints using 1 mm thick steel sheets. This material is used in the construction of light gauge steel frames for residential and commercial buildings. The authors discussed the results achieved for joints arranged in parallel and perpendicular to the load for specified joint spacing and for various combinations of joint types. The test results for rivet-clinched joints were compared with those for clinching alone. Joint strength can be increased by using rivet-clinching technology. Rivet-clinching allows for greater flexibility in sheet joining as the extensible die can be used in this new technology. Another new clinching method, namely die-less rivet-clinching, has also been introduced by Neugebauer et al. [167]. The die-less rivet-clinching works with a flat anvil as a counter tool. When joining materials by forming with a flat counter tool the crack-inducing tensile stresses in the bottom part are very low.

### 5.4. Laser-assisted clinching

Conventional clinching of steel substrates is currently limited to those with a tensile strength less than 800 N mm^–2^ and elongation at fracture more than 14%. High-strength steels can be clinched if the ductility at the site of the joint can be improved by localized heating. Reich et al. [212] proposed a laser assisted clinching method to solve this problem. With this new method, the overlap section is heated locally by a laser during the clinching process. As long as the heating is confined to the joint area, the properties of the material away from the joint should be unchanged by the joining process. In this context, Osten et al. [213] investigated the behavior of press hardened steel 22MnB5 when subjected to short-time heating. The mechanical properties of samples were studied by thermo-mechanical analysis in a deformation dilatometer. The results showed, for the first time, the feasibility of laser-assisted clinching between sheets of 22MnB5 press-hardened steel.

## 6. Modified clinching processes

Conventional clinching technology has no thermal effect on the materials being joined and does not introduce complementary joining elements such as rivets that add weight to the structure. On the other hand, the process causes a protrusion jutting out of the sheet plane on the die side. For this reason, conventional clinching cannot be used for visible and functional surfaces such as automotive body shells and sliding parts [56]. Also, access to both sides of the joint is essential and the technique cannot be used on brittle substrates. To overcome these problems, some modified clinching methods have been developed.

### 6.1. Flat clinching

Flat clinching was introduced in the 1990s [214]. In this process, the joined sheets are deformed after press clinching by being sandwiched between a punch and a flat die. This creates a new joint configuration which is flat on the bottom surface. Because at the end of the process one surface is flat, the technology can be applied even on functional surfaces. In a further variation, the second step was performed by pressing the joint between two flat dies and this has been shown to be very effective. The authors carried out FE simulations to optimize the process and the results for joint strength were verified by experimental tensile tests. Some joints were cut to show the changing of the contact line and how its characteristics influenced the bonding performance.

Wen et al. [19,215] proposed a new clinching method that involved compressing the joint with a pair of contoured tools. This compression controlled the local plastic deformation of the joint, reducing the height of the protrusion. The clinching, reshaping, and separation steps in the process were studied using two sheets of 6063 aluminum alloy (AA6063) with the thickness of 0.8 mm each. The values for the geometric parameters of the reshaping tools were optimized using the results of numerical simulation and orthogonal design so as to result in pre-determined pull-out strength. The connecting strengths of the clinched joints were compared before and after reshaping. The researchers were able to show that the joint protrusion resulting from the conventional clinching process can be reduced obviously by the compression step without decreasing the load-bearing capacity.

Another method for flat clinching was developed at the Chemnitz University of Technology [216,217]. The researchers found that the material flow during this flat clinching process results in interlocking within the total thickness of the sheets being joined. This creates a one-sided planar joint that has no die-side protrusion.

**Figure 16.**
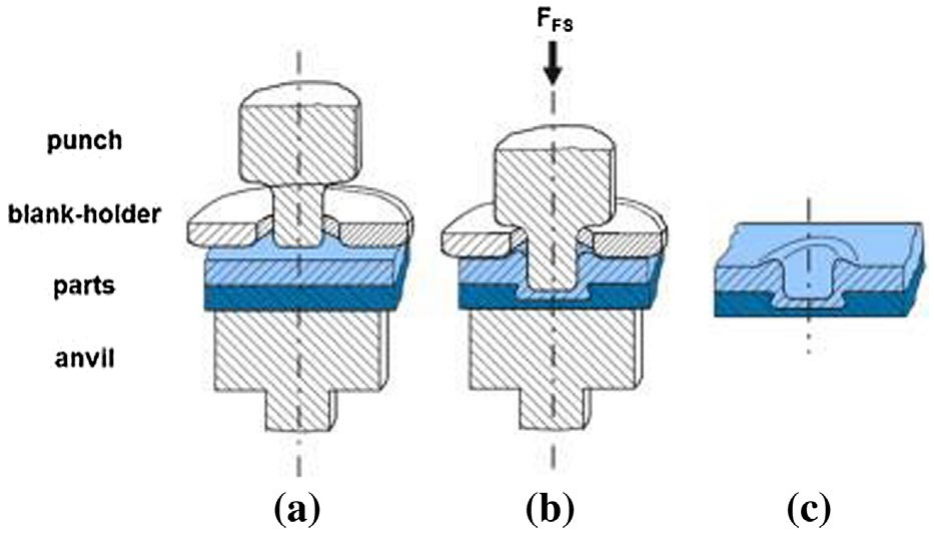
Schematic of flat clinching: (a) point location, (b) extruding deformation, (c) flat-clinched joint [179].

Flat-clinched joints can also be formed between sheets of wood-based composites made of different aluminum. Lüder et al. [179] conditioned wood materials to a specific moisture content and then flat-clinched them with sheets of aluminum. Joined specimens were relaxed under standard climatic conditions for 48 h and then subjected to cross-tension load tests at different moisture contents to determine the effect of moisture content on joint strength. Figure 16 shows the process of joining a flat-clinch connection [179]. The joining process was modeled using a FE method so as to ascertain the material flow during the flat clinching process [185]. The researchers carried out systematic numerical analysis and used the results to optimize the values of the parameters that influence the formation of the interlock and the neck thickness and therefore the load-bearing capacity of the joint. The validity of the simulation models was confirmed by analysis of experimental tests.

As mentioned earlier in this paper, joining magnesium alloys by forming is restricted by the properties of magnesium at room temperature. Parts being joined must be heated to at least 220°C before the clinching process results in joints without cracks. Neugebauer et al. [167–169,218] conducted a systematic investigation and presented an improved flat clinching method with important benefits when joining magnesium. In this method, the anvil is heated and the transfer of heat energy from the anvil to the parts being joined is relatively rapid. This makes it possible to decrease the pre-heating time from about 5 s to 1 s or less. The authors also carried out simulations using DEFORM software to investigate the impact that punch geometry has on the characteristics of the resulting flat clinched joints between various combinations of materials and component thicknesses. Limitations on the application of flat clinching method were also discussed. Contrary to conventional clinching, in flat clinching the counter tool cannot be used to control the material flow. Thus some restrictions exist concerning the combinations of material thicknesses. For clinching sheets with different thicknesses the sheet with the higher thickness should be used as the bottom sheet.

For increasing the load-bearing capacity of mechanical clinching, a height-reducing technique was investigated recently by Chen et al. [219,220]. A flat die and a bumped die (or pair of flat dies) were used to produce the height-reduced joint. The material of the protrusion flowed to the neck, which increased the load-bearing capacity of the joint by increasing the neck thickness. The cross-tensile strength, energy absorption, failure mode and geometrical parameter of the joint were investigated. The results show that the load-bearing capacity and energy absorption of the joint can be enhanced. The height-reducing method can increase the neck thickness with the decrease of the protrusion height. The height-reducing technique is effective for increasing the load-bearing capacity of the clinched joint. Chen et al. [221,222] also proposed a riveting technique to reshape the clinched joint. With this new technique, the protrusion can be compressed in a single stroke by a pair of flat dies. FE simulation and orthogonal design were used to optimize the geometrical parameters of the reshaping rivet.

### 6.2. Hydro-clinching

**Figure 17.**
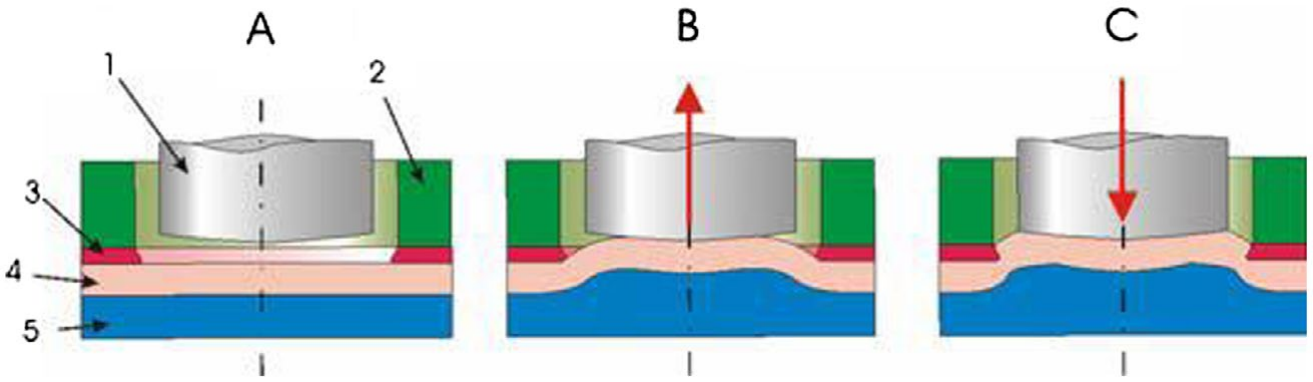
Operational sequence of the hydro-clinching [224].

Hydro-clinching is a new joining method involving a combination of hydro-forming and clinching that was proposed by Neugebauer et al. [223,224]. In hydro-clinching, a high pressure fluid is used as the die and this substitution extends the range of applications for clinching to areas not covered by standard clinching methods. For example, joining complex mechanical parts usually involves employing medium-based forming processes either because the insides of the parts are inaccessible, because the joining operation involves attaching parts or because handling of the parts being joined is difficult for some reason. Integrating clinching and hydro-forming processes can shorten assembly times and avoid the distortion of parts that might otherwise occur during subsequent thermal joining. Figure 17 shows the operational sequence of hydro-clinching [224]. The authors also discussed the potential and limits of this new method in comparison with alternative methods. The advantage of hydro-clinching is the decrease of the number of processing steps and the new design possibilities. The high accuracy of the hydro-clinching can be maintained by the cold joining technique, which represents a large advantage in relation to the welding techniques used so far.

### 6.3. Hole clinching

**Figure 18.**
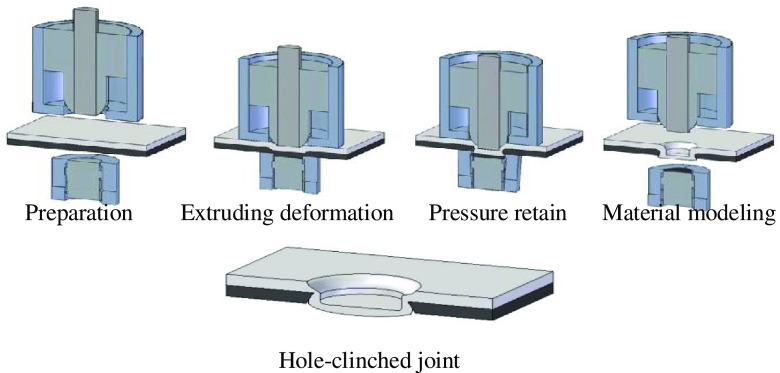
Hole-clinching process and hole-clinched joint [227].

For car manufacturers, joining dissimilar materials by mechanical joining is an important technique as it enables car bodies to be constructed from combinations of different materials. However, it is difficult to join ductile materials such as aluminum alloy to high-strength/low-ductility materials, such as carbon fiber reinforced plastic, hot-pressed steel and advanced high-strength steel, by conventional clinching. A new process called hole-clinching may find use in joining these materials without additional elements such as rivets [225]. In this single-stage process the die-side material is pre-punched by indirect hole-cutting and then the upper layer is formed into this hole. Figure 18 shows the hole-clinching process and the resulting hole-clinched joint. This variation of conventional clinching has the potential to greatly enlarge the applicability of clinching even if hot stamped steels are positioned on the die-side. The technique has been studied by Busse et al. [226,227] and Müller et al. [228] in relation to non-welded joining of high strength steel and aluminum sheets. FE analysis was used to explore and confirm different tool concepts and later on for the selection of appropriate values for the process parameters. Geometric optimization was determined by numerical simulation, and the feasibility of forming clinched joints by this method was confirmed by conducting physical experiments. Joined specimens were subjected to shear tensile and quasi-static cross tensile loads to determine the load-bearing capacities of the joints. Detailed micrographs were also prepared.

The applicability of hole-clinching techniques was elaborated by Lee et al. [229–232]. These systematic studies were aimed at developing new hole-clinching processes for combinations of materials that are otherwise difficult to join. New tools were designed in terms of the forming volume and the load-bearing capacity required. The authors analyzed the results of FE simulations and conducted physical experiments to verify the practicality of the hole-clinching process. Single-lap joint specimens were subjected to shear tests in order to evaluate the joint strength. Results showed that the hole-clinched joints showed comparable joint strength regardless of the material combinations thus demonstrating applicability of hole-clinching as a method for joining dissimilar materials.

### 6.4. Injection clinching

**Figure 19.**
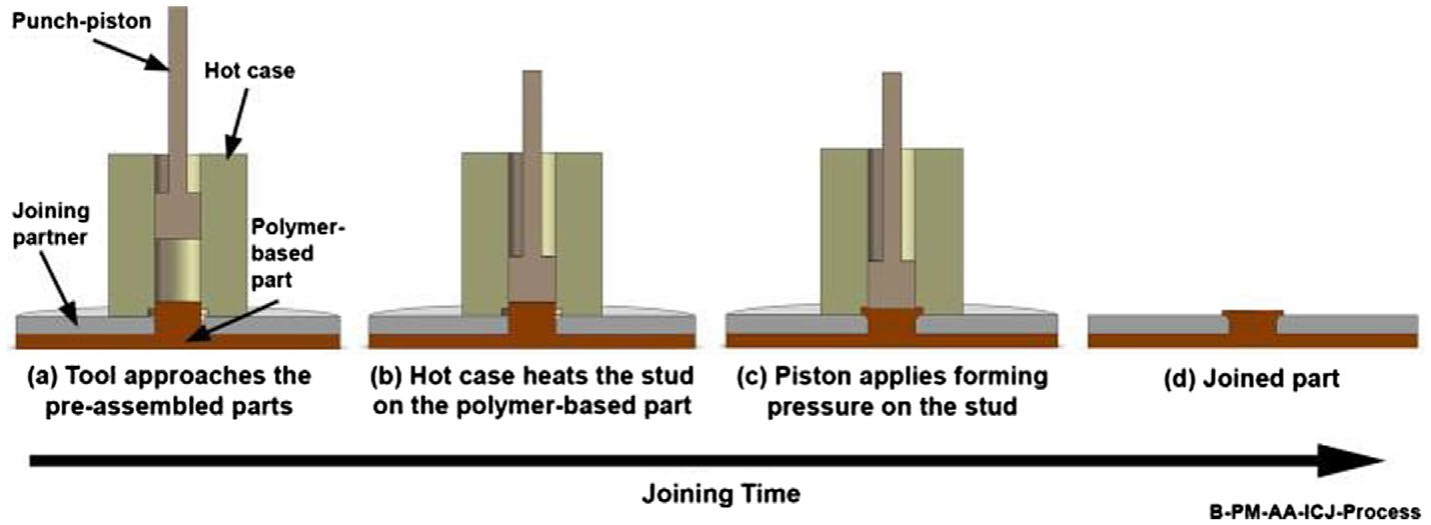
Steps of injection clinching process [178].

Applications in the transportation industry that use multi-material structures have become more numerous in recent years. The current and developing technologies for joining parts in such complex structures have the potential to result in significant engineering and performance improvement. A new process called injection clinching has been developed for joining hybrid polymer–metal structures [177]. Injection clinching is based on staking, adhesive bonding and injection molding technologies and provides spot joints with mechanical anchoring of the polymeric material in a specially designed cavity in a metallic part. The steps involved in the process are shown in Figure 19.

**Figure 20.**
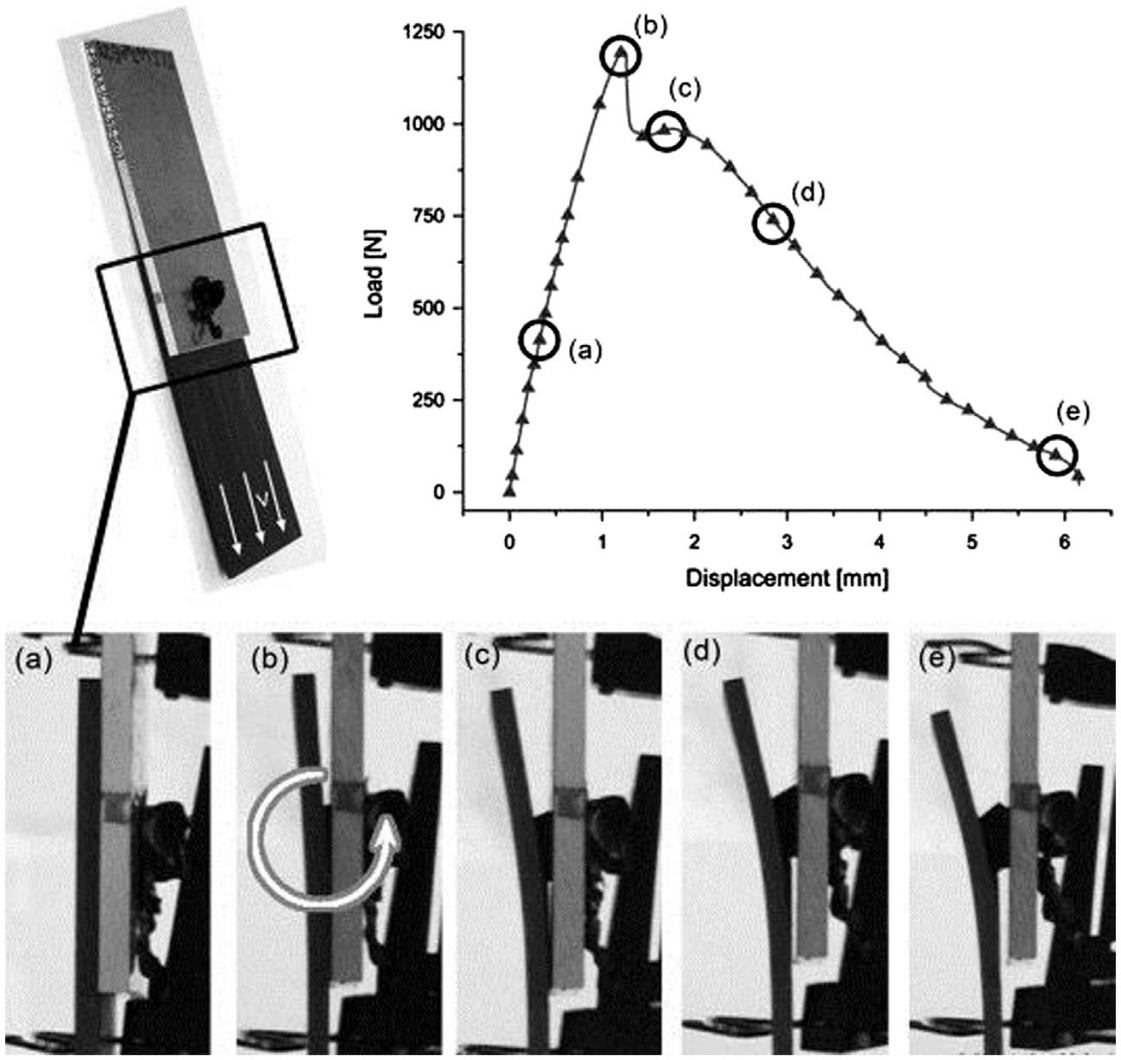
Typical behavior of an injection clinched joint when tested for lap shear strength (a)–(e) stages observed during the test [178].

**Figure 21.**
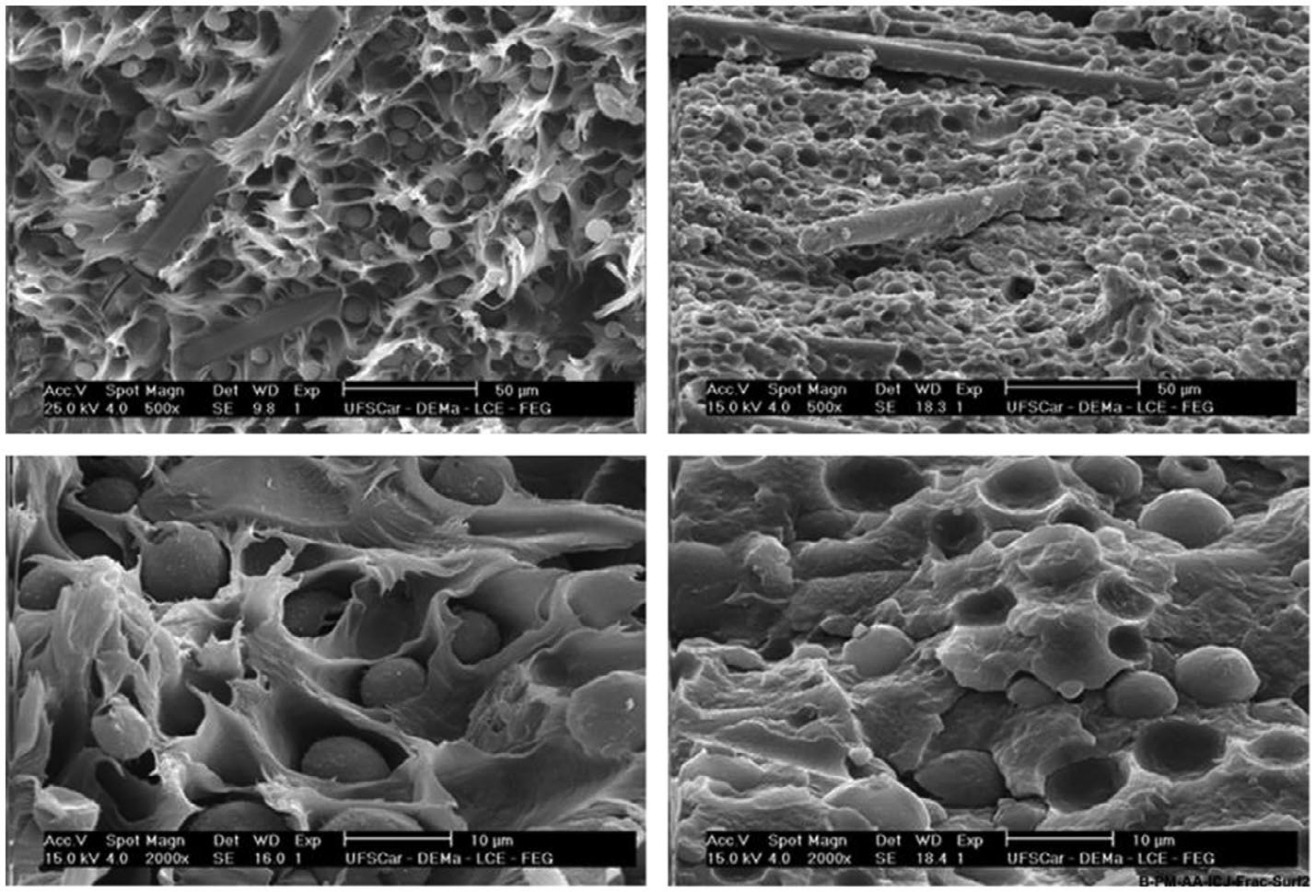
Fracture surface of PA66-GF composite after injection clinching joining (ICJ): (a) rivet pull-out failure, ductile: condition 60C-PA66 (composite dried for 24 h at 60°C) and (b) net tension, brittle failure: condition 120C-PA66 (composite dried for 24 h at 120°C) [178].

A study on the feasibility of joint formation involving a polyamide thermoplastic composite and aluminum was reported by Abibe et al. [177]. The authors addressed the mechanical, micro-structural, and thermal properties of injection clinched joints. In another paper, Abibe et al. [178] reported investigations into ways of exploiting the mechanical behavior of overlap joints produced by injection clinching and assessed their applicability. The measurements of lap-shear strength and *in situ* strain distribution were made using X-ray computer micro-tomography, scanning electron microscopy and optical microscopy. Different failure modes occurred, depending on the joining conditions. Net tension failure was brittle and catastrophic, while rivet pull-out resulted in a more desirable slow ductile failure mode (see Figure 20). The joint strengths ranged from 35.9% to 88.5% of strength of the base material under tensile stress. The fracture surface of PA66-GF composite of ICJ joint is shown in Figure 21.

## 7. Outlook

A good understanding of clinching processes and the behavior of clinched joints is essential to ensure the efficiency, safety and reliability of such joints in practice. Experimental and numerical studies of clinching processes can provide realistic predictions of the main variables affecting both the clinching processes and the in-service characteristics of the resulting joints. Knowledge gained from these studies accelerates the subsequent design processes and reduces the cost of optimizing the variables. However, several important issues need be addressed.

A high quality, low cost, predictable, reliable and repeatable clinching process will need to be developed if clinching is to be used in mass production. At present there are no agreed standards covering the clinching process. The most common configuration tested so far is the single-lap joint. This is favored because it is simple and quick to fabricate. But single lap joints are not necessarily appropriate for evaluating joint behavior because they comprise only a fraction of the joint types used in practice. To illustrate this point, substrate bending is affected by eccentricity in the load path and this adversely affects the mechanical behavior of single-lap clinched joints under laboratory conditions. Thus the geometry of specimens and the clinching processes need to be standardized so that test performance better reflects the in-service performance.

FE analysis is the most frequently used approach used in the simulation of clinching. FE simulations have contributed to a deeper understanding of the parameters determining the outcome of clinching processes, thereby enabling designers to estimate the mechanical properties of clinched joints and to predict failure mechanisms. In any particular case, the FE model must include all the relevant information from the clinching process. This means that accurate and reliable modeling of the process is still very difficult. Moreover, during the clinching process, a workpiece normally undergoes large deformation and this causes severe distortion of the elements in a FE analysis. The amount of distortion in elements may lead to instability in the numerical calculation and divergence to a nonlinear solution in FE analysis. Other numerical methods such as the finite difference method, particle method, etc., can be used to avoid these problems with difficult or troublesome clinching processes. A suitable numerical method is useful for establishing computational clinching process models that are effective, reliable and bi-directional in nature. Models for simulating mechanical clinching process are still under development and much work is still needed.

The effect of process parameters on clinched joint quality is well established only for aluminum alloys and ferroalloys. For some lightweight alloys, such as titanium alloys and magnesium alloys, clinching is restricted by the limited ductility of the materials at room temperature. In order to form joints without cracks, the material at the joint site must be heated to an appropriate temperature. New methods for localized heating are being developed to improve ductility at the site of the joint while minimizing or preventing adverse effects elsewhere in the materials being joined. Examples of these new methods include laser-assisted clinching and ultrasonic-assisted clinching. The mechanical properties of the materials during short-time heat treatment can be investigated by thermo-mechanical analysis in a deformation dilatometer. It is commonly believed that the addition of these new joining techniques in clinched joints is beneficial but it is not yet clear whether they have negative effects on the mechanical properties of the resulting clinched joints. Little work has been done in this area and consequently there is a distinct lack of information regarding the practical use of these hybrid clinching techniques.

The clinching process involves very high shear strains. The high strain rates are sufficient to cause micro-structural changes in some work hardened alloys. The mechanisms of micro-structural evolution also vary in different alloys. Thus it is still early for the application of clinching to materials other than aluminum alloys and ferroalloys. While preliminary studies showed that clinching is a potential technique for obtaining reliable joints in some new light alloys [233–238], there is an obvious need to gain a full understanding of the hybrid clinching processes and their interactions which determine micro-structures. This is a major challenge for understanding of the joining mechanisms and failure mechanisms of clinching for sheet materials.

Clinching is already used industrially for joining aluminum alloys and ferroalloys in airplanes and car manufacturing. With hybrid clinching and modified clinching processes, the progress made in joining of other advanced materials such as many lightweight alloys and manifold composites will make the mass production of light transportation systems possible and hence a significant reduction in energy consumption will be achieved.

## 8. Summary

The use of clinching enables greater and more targeted use of different lightweight materials in many engineering applications. Careful use of the technology enables manufacturers to improve the mechanical properties of structures and reduce their weight. The information on joining processes obtained from fundamental research leads to improvements in tools and process design, thereby reducing costs and improving the quality of the products. The powerful combination of computer simulation and laboratory tests can also be used to set the initial parameters for further studies of the mechanical properties of joints and structures, static and fatigue analysis, crash analysis, prediction of assembly dimensions, etc.

In recent years there have been exciting developments in clinching techniques and the successful industrial use of the technology to join sheet materials that are difficult to weld. However, the technology is still in its infancy and so far much of the development of the clinching process for each new application has been largely empirical. Scientific knowledge-based studies are, and will continue to be, of significant help in understanding the clinching process and its application in solving real-world engineering problems.

In this paper, the research activities and the progress made to date in clinching are reviewed. Some important issues such as tool design and process parameters are highlighted. The joinability of new lightweight sheet materials by clinching is introduced. The hybrid clinching processes and modified clinching processes are described. In addition, several unaddressed issues in the clinching of sheet materials are identified.

## Disclosure statement

No potential conflict of interest was reported by the author.
